# Distributed Density Estimation Based on a Mixture of Factor Analyzers in a Sensor Network

**DOI:** 10.3390/s150819047

**Published:** 2015-08-05

**Authors:** Xin Wei, Chunguang Li, Liang Zhou, Li Zhao

**Affiliations:** 1 College of Telecommunications and Information Engineering, Nanjing University of Posts and Telecommunications, Nanjing 210003, China; E-Mail: liang.zhou@njupt.edu.cn; 2 Department of Information Science and Electronic Engineering, Zhejiang University, Hangzhou 310027, China; E-Mail: cgli@zju.edu.cn; 3 School of Information Science and Engineering, Southeast University, Nanjing 210096, China; E-Mail: zhaoli@seu.edu.cn

**Keywords:** distributed density estimation, mixture of factor analyzers, mixture of Student’s *t*-factor analyzers, sensor network

## Abstract

Distributed density estimation in sensor networks has received much attention due to its broad applicability. When encountering high-dimensional observations, a mixture of factor analyzers (MFA) is taken to replace mixture of Gaussians for describing the distributions of observations. In this paper, we study distributed density estimation based on a mixture of factor analyzers. Existing estimation algorithms of the MFA are for the centralized case, which are not suitable for distributed processing in sensor networks. We present distributed density estimation algorithms for the MFA and its extension, the mixture of Student’s *t*-factor analyzers (M*t*FA). We first define an objective function as the linear combination of local log-likelihoods. Then, we give the derivation process of the distributed estimation algorithms for the MFA and M*t*FA in details, respectively. In these algorithms, the local sufficient statistics (LSS) are calculated at first and diffused. Then, each node performs a linear combination of the received LSS from nodes in its neighborhood to obtain the combined sufficient statistics (CSS). Parameters of the MFA and the M*t*FA can be obtained by using the CSS. Finally, we evaluate the performance of these algorithms by numerical simulations and application example. Experimental results validate the promising performance of the proposed algorithms.

## 1. Introduction

Sensor networks are composed of tiny, intelligent sensor nodes that are deployed over a geographic region. This type of network has a broad range of applications, such as environmental monitoring, precision agriculture and military surveillance [1,2,3]. Distributed estimation over sensor networks is to estimate some parameters of interest through local computation and information exchange among neighbor nodes. Compared to centralized estimation, it does not need to send observations collected by all of the sensors to a powerful central node, so the complexity and resource consumption can be reduced. Furthermore, distributed estimation is more flexible and robust to node and/or link failure [2,4]. Recently, many distributed estimation algorithms have been proposed, such as distributed LMS [5], distributed recursive least squares (RLS) [6], distributed source location [7], distributed power allocation [8], distributed sparse estimation [9,10], distributed information theoretic learning [11] and distributed Gaussian process regression [12].

Mixture of Gaussians (GMM) is a flexible and powerful probabilistic modeling tool for density estimation. It has been used in several areas, such as pattern recognition, computer vision, signal and image analysis and machine learning. When estimating parameters in the GMM by the maximum likelihood criterion, the expectation maximization (EM) algorithm [13,14] is usually adopted. It iteratively performs the expectation step (E-step) to calculate the conditional expectations of unobserved/hidden variables and runs the maximization step (M-step) to estimate parameters of data distributions based on the result of the E-step. However, when the dimension of observations is high, the fitting performance of the GMM deteriorates or even the associated EM algorithm cannot work [15]. The main reason is that the GMM cannot realize dimensionality reduction, which is to compress highly-correlated components of observations. In this case, a mixture of factor analyzers (MFA) [16,17] can be considered. The MFA combines local factor analysis in a form of a finite mixture. As factor analysis can describe variability among high-dimensional observations in terms of potentially low-dimensional latent factors, the MFA can carry out dimensionality reduction simultaneously when finishing specific tasks. Moreover, in order to process the non-normality of data or outliers, normal distributions in the MFA can be replaced by Student’s *t*-distributions, obtaining the mixture of Student’s *t*-factor analyzers (M*t*FA) [18,19]. Therefore, the MFA and its extension M*t*FA are effective tools for processing high-dimensional observations [20]. They have been successfully applied in the domains of signal processing [21,22], bioinformatics [23,24] and other applied fields.

In sensor networks, GMM has been introduced for density estimation of observations [25,26,27,28,29,30,31,32]. The estimation process for the GMM needs to be realized by distributed EM algorithms. According to the way by which nodes communicate with each other, distributed EM algorithms can be classified into the incremental type [25,26], the consensus type [27] and the diffusion type [28,29,30,31,32]. In the incremental scheme [25,26], a long way from the first node to last node of the pre-selected path is needed. When any node along the path fails, reliability problem may happen. In the consensus-based distributed EM algorithm for the GMM [27], a consensus filter, by which global statistics for each node is achieved, is carried out between the E-step and the M-step at each iteration. The objective is to obtain the same estimations for all nodes at each iteration. In the diffusion type of distributed estimation for the GMM, each node exchanges information only with its neighbors through a diffusion cooperative protocol. Good performance is obtained while communication overhead is kept low [2,4]. In this paper, we focus on a diffusion type of distributed estimation. Among previous studies, a distributed model order and parameter estimation algorithm for the GMM was proposed in [28]. Moreover, algorithm performance was analyzed. In [29], a diffusion-based EM algorithm was presented for distributed estimation in unreliable sensor networks. In this scenario, some nodes may be subject to data failures and report only noise. The aim of the algorithm was to achieve the optimal performance within the whole range of SNRs. In [30], information diffusion and averaging were considered and performed simultaneously. In [31], an adaptive diffusion scheme was proposed. In [32], the performance of the diffusion-based EM algorithm was analyzed. It could be considered as a stochastic approximation method [33] to find the maximum likelihood estimation of the GMM.

As the MFA can handle high-dimensional observations, which are also usually encountered in sensor networks, in this paper, we propose distributed density estimation algorithms for the MFA and its extension M*t*FA. We represent these two algorithms as D-MFA and D-M*t*FA, respectively. Specially, for each node in the sensor network, we define an objective function as the linear combination of local log-likelihoods, whose combination weights are determined by the number of observations in the corresponding neighbor nodes. After local sufficient statistics are computed, the current node calculates its combined sufficient statistics by a linear weighed combination of these local sufficient statistics from nodes in its neighborhood set. Finally, parameters of the MFA and M*t*FA are updated using the combined sufficient statistics. Apart from the distributed processing of the MFA and the M*t*FA in this paper, there are two other differences from the existing algorithms. First, in the relevant algorithms [25,27,28], mixing proportions in the GMM are different at each node, whereas means and covariances are the same throughout the network. On the contrary, in this paper, all of the parameters in the MFA or the M*t*FA are the same throughout the network. Using this design, distributed clustering and classification can be done in arbitrary nodes after the estimation process finishes. Second, for each node, the objective function is directly defined. The combination of weights in the objective function is effectively designed.

The rest of this paper is organized as follows. In Section 2, a brief overview of the MFA and the M*t*FA are provided. In Section 3, the D-MFA algorithm and D-M*t*FA algorithm are formulated. In Section 4, numerical simulations for the synthetic observations are performed to illustrate the effectiveness and advantages of the proposed algorithms. Moreover, the application of these algorithms to distributed clustering is also presented. Finally, conclusions are drawn in Section 5.

The acronyms mentioned in this paper are listed in the following.
Acronym list:
GMMGaussian mixture modelEMexpectation maximizationE-stepexpectation stepM-stepmaximization stepMFAmixture of factor analyzersM*t*FAmixture of Student’s *t*-factor analyzersD-MFAdistributed density estimation algorithm for the MFAD-M*t*FAdistributed density estimation algorithm for the M*t*FACSScombined sufficient statisticsLSSlocal sufficient statisticsS-MFAstandard EM algorithm for the MFAS-M*t*FAstandard EM algorithm for the M*t*FANC-MFAnon-cooperation MFANC-M*t*FAnon-cooperation M*t*FAD-GMMdistributed density estimation algorithm for the GMMD-*t*MMdistributed density estimation algorithm for the Student’s*t*-mixture model

## 2. Preliminaries: MFA and M*t*FA

### 2.1. Mixture of Factor Analyzers

Let the observed dataset be $Y=\{y_{1},...,y_{N}\}$. In the MFA, it assumes that each *p*-dimensional data vector $y_{n}$ is generated as:

(1) $$y_{n}=\mu_{i}+A_{i}u_{n}+e_{ni}\;\;\;\text{ with prob. }\;\pi_{i}\;\left(i=1,...,I\right)$$

where *I* is the number of mixing components. The corresponding *q*-dimensional (q<p) factor $u_{n}∼𝒩\left(u_{n}|0,I_{q}\right)$ is independent of the $e_{ni}∼𝒩\left(e_{ni}|0,D_{i}\right)$, where $D_{i}$ is a p×p diagonal matrix. The parameter $\mu_{i}$ is the mean of the *i*-th analyzer, and $A_{i}$ (p×q) is the linear transformation known as the factor loading matrix. The so-called mixing proportions $\pi_{i}\;\left(i=1,...,I\right)$ are nonnegative and sum to one. The standard EM algorithm for the MFA is given in [15,16].

### 2.2. Mixture of Student’s *t*-Factor Analyzers

Since the MFA adopts the normal family for the distributions of the errors and the latent factors, it is sensitive to outliers. An obvious way to improve the robustness of this model for observations having longer tails than normal is using the *t*-family of elliptically-symmetric distributions. Therefore, the M*t*FA has been proposed in [18]. In the M*t*FA, it assumes that *p*-dimensional data vector $y_{n}$ is generated in the same way as that in the MFA, as shown in Equation (1). However, the distributions of *q*-dimensional (q<p) factor $u_{n}$ and noise $e_{ni}$ are $t(u_{n}|0,I_{q},\nu_{i})$ and $t(e_{ni}|0,D_{i},\nu_{i})$, respectively. In the above Student’s *t* distributions, $\nu_{i}$ is called the degree of freedom that controls the length of the tails of the distributions. With this modification, the M*t*FA is more robust to outliers and can process the non-normality of observations in a better way [23]. In essence, $t(u_{n}|0,I_{q},\nu_{i})$ and $t(e_{ni}|0,D_{i},\nu_{i})$ can be respectively regarded as average Gaussian scale distributions $𝒩(u_{n}|0,I_{q}/w_{ni})$ and $𝒩(e_{ni}|0,D_{i}/w_{ni})$ with the Gamma distributed precision scalar $w_{ni}$, that is:

$$\begin{array}{ccc} t(u_{n}|0,I_{q},\nu_{i}) & = & \int dw_{ni}𝒩\left(u_{n}|0,I_{q}/w_{ni}\right)𝒢\left(w_{ni}|\nu_{i}/2,\nu_{i}/2\right)\\ t(e_{ni}|0,D_{i},\nu_{i}) & = & \int dw_{ni}𝒩\left(e_{ni}|0,D_{i}/w_{ni}\right)𝒢\left(w_{ni}|\nu_{i}/2,\nu_{i}/2\right) \end{array}$$

where 𝒢(·) denotes the Gamma distribution. The standard EM algorithm for the M*t*FA is given in [14,18].

## 3. Distributed Estimation Algorithms for the MFA and M*t*FA

### 3.1. Network Model and Objective Function

Consider a sensor network with *M* nodes. The *m*-th node has $N_{m}$ data observations $Y_{m}={\left\{y_{m,n}\right\}}_{n=1,...,N_{m}}\;\left(m=1,...,M\right)$, and $y_{m,n}$ denotes the *n*-th observation in node *m*. The distribution of each *p*-dimensional observation $y_{m,n}$ is modeled by the MFA, defined in Equation (1). It is noted that the factor associated with $y_{m,n}$ here is represented as $u_{m,n}$. The parameter set of the MFA is $\Theta ={\left\{\pi_{i},\mu_{i},A_{i},D_{i}\right\}}_{i=1,...,I}$, which is to be estimated.

**Figure 1:**
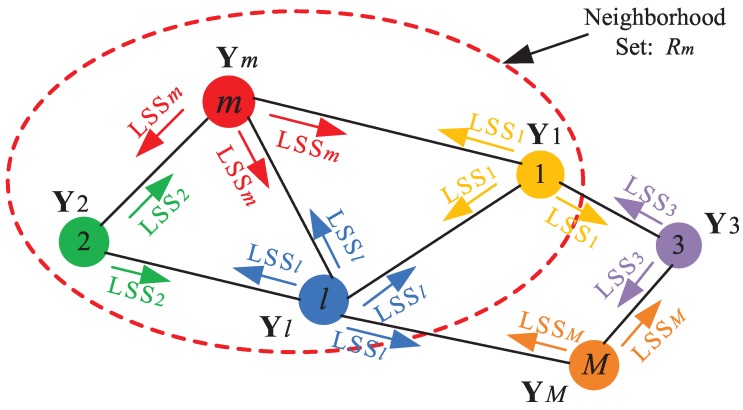
A sensor network consists of a collection of cooperating nodes. Node *m* only exchanges information (e.g., local sufficient statistics (LSS) in the proposed D-MFA and D-M*t*FA algorithms) with nodes in $R_{m}$.

The network topology is described by a graph. Let *W* denote the distance that a node can communicate via wireless radio links. Nodes *m* and *l* are connected if the Euclidean distance $d_{m,l}$ between *m* and *l* is less than or equal to *W*. Moreover, a graph is connected if for any pair of nodes (m,n), there exists a path from *m* to *n*. The neighborhood set of node *m*, denoted by $R_{m}$, is defined as the one-hop neighbors of node *m* (including *m* itself). For example, in Figure 1, the dashed circle represents the neighborhood set of node *m*, containing Node 1, Node 2, node *l* and node *m* itself.

In order to design the D-MFA and the D-M*t*FA algorithms, the objective functions should be carefully specified at first. Here, we take node *m* in the sensor network for example. We define the objective function ${ℱ}_{m}\left(\Theta \right)$ of the MFA at node *m* as a linear combination of local log-likelihoods $\log p(Y_{l}|\Theta )$ associated with nodes *l* in its neighborhood $R_{m}$.

(2) $${ℱ}_{m}\left(\Theta \right)=\sum_{l\in R_{m}}c_{lm}\cdot \log p\left(Y_{l}|\Theta \right)=\sum_{l\in R_{m}}c_{lm}\sum_{n=1}^{N_{l}}\log\left(\sum_{i=1}^{I}\pi_{i}N\left(y_{l,n}|\mu_{i},A_{i},D_{i}\right)\right)$$

where ${\left\{c_{lm}\right\}}_{l\in R_{m}}$ are some non-negative combination coefficients satisfying the condition $\sum_{l\in R_{m}}c_{lm}=\;1$, $c_{lm}=0$ if node $l\notin R_{m}$.

It should be emphasized that when defining ${ℱ}_{m}\left(\Theta \right)$, we consider two important factors. First, as node *m* can only communicate with its neighbors, it is reasonable to define ${ℱ}_{m}\left(\Theta \right)$ as a combination of local log-likelihoods $\log p\left(Y_{l}|\Theta \right),\;\left(l\in R_{m}\right)$. When estimating **Θ**, node *m* can make use of the information from nodes in $R_{m}$. Due to the effect of information diffusion, each node can obtain all information directly or indirectly from other nodes. Second, the contributions of different local log-likelihoods $\log p\left(Y_{l}|\Theta \right),\;\left(l\in R_{m}\right)$ for the estimation of **Θ** at node *m* may also be different. The combination coefficient $c_{lm}$ weights the importance of information flow from the node $l\;(l\in R_{m})$. Therefore, how to choose $c_{lm}$ is important. Here, we adopt a simple, but effective mechanism that $c_{lm}$ is determined by:

(3) $$c_{lm}=\frac{N_{l}}{\sum_{l^{\prime }\in R_{m}}N_{l^{\prime }}}$$

If node *l* has a larger number of observations $N_{l}$, the information from this node makes a larger contribution to obtain more accurate parameter estimations. Therefore, a larger combination coefficient $c_{lm}$ in Equation (3) can further make this contribution prominent. In the future, a more effective implementation, such as an adaptive strategy [4], can be considered to determine these combination coefficients better.

### 3.2. Distributed Density Estimation Algorithm for the MFA

After the objective functions ${ℱ}_{m}\left(\Theta \right)\;\left(m=1,...,M\right)$ have been determined, the next task is to estimate parameters **Θ** in the MFA by maximizing ${ℱ}_{m}\left(\Theta \right)$. For node *l*, an *I*-dimensional binary latent variable $z_{l,n}$, which is associated with $y_{l,n}$, is introduced. As the MFA is a mixture model, $z_{l,n,i}\left(=1\right)$ denotes that $y_{l,n}$ belongs to the *i*-th component of the MFA. The latent variables for node *l* in the neighborhood $R_{m}$ are $U_{l}={\left\{u_{l,n}\right\}}_{n=1,...,N_{l}}$, $Z_{l}={\left\{z_{l,n}\right\}}_{n=1,...,N_{l}}$, $\left(l\in R_{m}\right)$. Now, ${ℱ}_{m}\left(\Theta \right)\;\left(m=1,...,M\right)$ in Equation (2) can be expressed as:

$${ℱ}_{m}\left(\Theta \right)=\sum_{l\in R_{m}}c_{lm}\cdot \left\{\log\sum_{Z_{l}}\int dU_{l}\;p\left(Y_{l},Z_{l},U_{l}|\Theta \right)\right\}$$

where:

(4) $$p\left(Y_{l},Z_{l},U_{l}|\Theta \right)=p\left(Z_{l}|\Theta \right)p\left(U_{l}|Z_{l},\Theta \right)p\left(Y_{l}|Z_{l},U_{l}\Theta \right)$$

The three conditional probabilities in Equation (4) are:

$$\begin{array}{ccc} p(Z_{l}|\Theta ) & = & \prod_{n=1}^{N_{l}}\prod_{i=1}^{I}\pi_{i}^{z_{l,n,i}}\\ p(U_{l}|Z_{l},\Theta ) & = & \prod_{n=1}^{N_{l}}\prod_{i=1}^{I}𝒩{\left(u_{l,n}|0,I_{q}\right)}^{z_{l,n,i}}\\ p(Y_{l}|Z_{l},U_{l},\Theta ) & = & \prod_{n=1}^{N_{l}}\prod_{i=1}^{I}𝒩{\left(y_{l,n}|\mu_{i}+A_{i}u_{l,n},D_{i}\right)}^{z_{l,n,i}} \end{array}$$

Here, we derive the distributed estimation algorithm with the aid of the standard EM algorithm [13]. First, we introduce two distributions $q(Z_{l})$ and $q(U_{l}|Z_{l})$ defined over the latent variables. For any choice of $q(Z_{l})$ and $q(U_{l}|Z_{l})$, the following decomposition holds:

(5) $${ℱ}_{m}\left(\Theta \right)=\sum_{l\in R_{m}}c_{lm}\cdot ℒ\left(q_{l},\Theta \right)+\sum_{l\in R_{m}}c_{lm}\cdot \mathrm{KL}(q_{l}\left|\right|p_{l})$$

where:

$$\begin{array}{ccc} ℒ(q_{l},\Theta ) & = & \sum_{Z_{l}}q\left(Z_{l}\right)\int dU_{l}q\left(U_{l}|Z_{l}\right)\log\left\{\frac{p(Y_{l},Z_{l},U_{l}|\Theta )}{q\left(Z_{l}\right)q\left(U_{l}|Z_{l}\right)}\right\}\\ \mathrm{KL}(q_{l}||p_{l}) & = & -\sum_{Z_{l}}q\left(Z_{l}\right)\int dU_{l}q\left(U_{l}|Z_{l}\right)\log\left\{\frac{p\left(Z_{l}|Y_{l},\Theta \right)p\left(U_{l}|Z_{l},Y_{l},\Theta \right)}{q\left(Z_{l}\right)q\left(U_{l}|Z_{l}\right)}\right\}\;\; \end{array}$$

The verification of log-likelihood decomposition can be found in [34]. As ${ℱ}_{m}\left(\Theta \right)$ as a combination of local log-likelihoods, the whole decomposition can also be expressed by a combination of local log-likelihood decompositions, as shown in Equation (5).

Moreover, $\mathrm{KL}(q_{l}||p_{l})$ in Equation (5) is the Kullback–Leibler divergence between $q\left(Z_{l}\right)q\left(U_{l}|Z_{l}\right)$ and $p\left(Z_{l}|Y_{l},\Theta \right)p\left(U_{l}|Z_{l},Y_{l},\Theta \right)$, which satisfies $\mathrm{KL}(q_{l}||p_{l})\geq 0$. Therefore, it can be seen from Equation (5) that $\sum_{l\in R_{m}}c_{lm}\cdot ℒ\left(q_{l},\Theta \right)\leq {ℱ}_{m}\left(\Theta \right)$. In other words, $\sum_{l\in R_{m}}c_{lm}\cdot ℒ\left(q_{l},\Theta \right)$ is a lower bound on ${ℱ}_{m}\left(\Theta \right)$. As direct maximization of the ${ℱ}_{m}\left(\Theta \right)$ is difficult, it can be solved by the maximization of this lower bound instead.

Suppose that the parameters estimated in the last iteration are $\Theta^{\mathrm{old}}={\left\{\pi_{i}^{\mathrm{old}},\mu_{i}^{\mathrm{old}},A_{i}^{\mathrm{old}},D_{i}^{\mathrm{old}}\right\}}_{i=1,...,I}$. In the first stage, the lower bound $\sum_{l\in R_{m}}c_{lm}\cdot ℒ\left(q_{l},\Theta^{\mathrm{old}}\right)$ is maximized with respect to $q\left(Z_{l}\right)q\left(U_{l}|Z_{l}\right)$ while holding $\Theta^{\mathrm{old}}$ fixed. From Equation (5), this maximum can be achieved when $\mathrm{KL}(q_{l}||p_{l})=0$. In other words, $q\left(Z_{l}\right)q\left(U_{l}|Z_{l}\right)=p\left(Z_{l}|Y_{l},\Theta^{\mathrm{old}}\right)p\left(U_{l}|Z_{l},Y_{l},\Theta^{\mathrm{old}}\right)$. Therefore, two conditional distributions, $p(Z_{l}|Y_{l},\Theta^{\mathrm{old}})$ and $p(U_{l}|Z_{l},Y_{l},\Theta^{\mathrm{old}})$, should be computed.

Concretely, for node *l* ($l\in R_{m}$), $p(Z_{l}|Y_{l},\Theta^{\mathrm{old}})$ can be calculated by:

$$p\left(Z_{l}|Y_{l},\Theta^{\mathrm{old}}\right)=\prod_{n=1}^{N_{l}}\prod_{i=1}^{I}p\left(z_{l,n,i}|y_{l,n}\right)$$

where:

(6) $$p\left(z_{l,n,i}|y_{l,n}\right)=\frac{\pi_{i}^{\mathrm{old}}N\left(y_{l,n}|\mu_{i}^{\mathrm{old}},A_{i}^{\mathrm{old}}{\left(A_{i}^{\mathrm{old}}\right)}^{T}+D_{i}^{\mathrm{old}}\right)}{\sum_{i^{\prime }=1}^{I}\pi_{i^{\prime }}^{\mathrm{old}}N\left(y_{l,n}|\mu_{i^{\prime }}^{\mathrm{old}},A_{i^{\prime }}^{\mathrm{old}}{\left(A_{i^{\prime }}^{\mathrm{old}}\right)}^{T}+D_{i^{\prime }}^{\mathrm{old}}\right)}$$

Moreover, $p(U_{l}|Z_{l},Y_{l},\Theta^{\mathrm{old}})$ should also be obtained by:

(7) $$\begin{array}{c} p\left(U_{l}|Z_{l},Y_{l},\Theta^{\mathrm{old}}\right)=\prod_{n=1}^{N_{l}}\prod_{i=1}^{I}p\left(u_{l,n}|y_{l,n},z_{l,n,i}\right)=\prod_{n=1}^{N_{l}}\prod_{i=1}^{I}N\left(u_{l,n}|{\bar{u}}_{l,n,i},\Omega_{i}\right) \end{array}$$

The mean ${\bar{u}}_{m,n,i}$ and covariance $\Omega_{i}$ are:

(8) $$\begin{array}{ccc} {\bar{u}}_{l,n,i} & = & g_{i}^{T}\left(y_{l,n}-\mu_{i}^{\mathrm{old}}\right)\\ \Omega_{i} & = & I_{q}-g_{i}^{T}A_{i}^{\mathrm{old}} \end{array}$$

where:

(9) $$g_{i}={\left[A_{i}^{\mathrm{old}}{\left(A_{i}^{\mathrm{old}}\right)}^{T}+D_{i}^{\mathrm{old}}\right]}^{-1}\cdot A_{i}^{\mathrm{old}}$$

is an intermediate variable introduced to simplify expressions in the following steps.

When the above two conditional distributions have been obtained, $q\left(Z_{l}\right)q\left(U_{l}|Z_{l}\right)$ is determined and held fixed, and the lower bound $\sum_{l\in R_{m}}c_{lm}\cdot ℒ\left(q_{l},\Theta \right)$ is maximized with respect to **Θ** to get new estimated $\Theta^{\mathrm{new}}$. This will cause the lower bound to increase, which will necessarily cause the corresponding ${ℱ}_{m}\left(\Theta \right)$ to increase.

Concretely, the current lower bound is expressed as:

(10) $$\begin{array}{ccc} \sum_{l\in R_{m}}c_{lm}ℒ\left(q_{l},\Theta \right) & = & \sum_{l\in R_{m}}c_{lm}\sum_{Z_{l}}p\left(Z_{l}|Y_{l},\Theta^{\mathrm{old}}\right)\int dU_{l}p\left(U_{l}|Z_{l},Y_{l},\Theta^{\mathrm{old}}\right)\\ & & \times \left\{\log p\left(Y_{l},Z_{l},U_{l}|\Theta \right)-\log\left[p\left(Z_{l}|Y_{l},\Theta^{\mathrm{old}}\right)p\left(U_{l}|Z_{l},Y_{l},\Theta^{\mathrm{old}}\right)\right]\right\} \end{array}$$

Discard the second logarithmic term unrelated to **Θ** in Equation (10); the objective function represented by $Q_{m}\left(\Theta \right)$ at node *m* is:

$$\begin{array}{ccc} Q_{m}\left(\Theta \right) & = & \sum_{l\in R_{m}}c_{lm}\sum_{n=1}^{N_{l}}\sum_{i=1}^{I}p\left(z_{l,n,i}|y_{l,n}\right)p\left(u_{l,n}|y_{l,n},z_{l,n,i}\right)\;\\ & & \times \left\{z_{l,n,i}\log\left[\pi_{i}N\left(u_{l,n}|0,I_{q}\right)N\left(y_{l,n}|\mu_{i}+A_{i}u_{l,n},D_{i}\right)\right]\right\} \end{array}$$

Now, parameters in **Θ** can be obtained by taking derivation of $Q_{m}\left(\Theta \right)$ with respect to **Θ**.

First, $\pi_{i}$ and $\mu_{i}$ are updated to:

(11) $$\pi_{i}=\frac{\sum_{l\in R_{m}}c_{lm}\sum_{n=1}^{N_{l}}\langle z_{l,n,i}\rangle }{\sum_{i^{\prime }=1}^{I}\sum_{l\in R_{m}}c_{lm}\sum_{n=1}^{N_{l}}\langle z_{l,n,i^{\prime }}\rangle }$$

and:

(12) $$\mu_{i}=\frac{\sum_{l\in R_{m}}c_{lm}\sum_{n=1}^{N_{l}}\langle z_{l,n,i}\rangle y_{l,n}}{\sum_{l\in R_{m}}c_{lm}\sum_{n=1}^{N_{l}}\langle z_{l,n,i}\rangle }$$

respectively. Subsequently, by performing derivation of $Q_{m}\left(\Theta \right)$ with respect to $A_{i}$, we have:

(13) $$A_{i}=\frac{\sum_{l\in R_{m}}c_{lm}\sum_{n=1}^{N_{l}}\langle z_{l,n,i}\rangle \left(y_{l,n}-\mu_{i}\right){\langle u_{l,n}\rangle }^{T}}{\sum_{l\in R_{m}}c_{lm}\sum_{n=1}^{N_{l}}\langle z_{l,n,i}\rangle \cdot \langle u_{l,n}u_{l,n}^{T}\rangle }$$

Finally, the expression of $D_{i}$ can be obtained in the same way,

(14) $$D_{i}=\mathrm{diag}\left\{\frac{\sum_{l\in R_{m}}c_{lm}\sum_{n=1}^{N_{l}}\langle z_{l,n,i}\rangle \left[\left(y_{l,n}-\mu_{i}\right){\left(y_{l,n}-\mu_{i}\right)}^{T}-A_{i}\langle u_{l,n}u_{l,n}^{T}\rangle A_{i}^{T}\right]}{\sum_{l\in R_{m}}c_{lm}\sum_{n=1}^{N_{l}}\langle z_{l,n,i}\rangle }\right\}$$

where diag{·} denotes the operator setting off-diagonal terms to zero. In Equations (11,12,13,14), $\langle z_{l,n,i}\rangle$ is the expectation of $z_{l,n,i}$ given by Equation (6). $\langle u_{l,n}\rangle$ and $\langle u_{l,n}u_{l,n}^{T}\rangle$ can be obtained from Equation (7), which are:

(15) $$\langle u_{l,n}\rangle ={\bar{u}}_{l,n,i}\;\;\mathrm{and}\;\;\langle u_{l,n}u_{l,n}^{T}\rangle =\Omega_{i}+{\bar{u}}_{l,n,i}{\bar{u}}_{l,n,i}^{T}$$

respectively. Substituting Equation (15) into Equations (13) and (14), we have:

(16) $$A_{i}=V_{i}g_{i}{\left(g_{i}^{T}V_{i}g_{i}+\Omega_{i}\right)}^{-1}$$

and:

(17) $$D_{i}=\mathrm{diag}\left\{V_{i}-A_{i}\left(g_{i}^{T}V_{i}g_{i}+\Omega_{i}\right)A_{i}^{T}\right\}$$

where:

(18) $$V_{i}=\frac{\sum_{l\in R_{m}}c_{lm}\sum_{n=1}^{N_{l}}\langle z_{l,n,i}\rangle \left(y_{l,n}-\mu_{i}\right){\left(y_{l,n}-\mu_{i}\right)}^{T}}{\sum_{l\in R_{m}}c_{lm}\sum_{n=1}^{N_{l}}\langle z_{l,n,i}\rangle }$$

From Equations (11), (12) and (16)–(18), we can see, when estimating parameters **Θ** at node *m*, that three combined sufficient statistics (CSS) must be obtained, represented as:

(19) $$\begin{array}{ccc} {\mathrm{CSS}}_{m}^{\left(1\right)}\left[i\right] & = & \sum_{l\in R_{m}}c_{lm}\cdot {\mathrm{LSS}}_{l}^{\left(1\right)}\left[i\right]\\ {\mathrm{CSS}}_{m}^{\left(2\right)}\left[i\right] & = & \sum_{l\in R_{m}}c_{lm}\cdot {\mathrm{LSS}}_{l}^{\left(2\right)}\left[i\right]\\ {\mathrm{CSS}}_{m}^{\left(3\right)}\left[i\right] & = & \sum_{l\in R_{m}}c_{lm}\cdot {\mathrm{LSS}}_{l}^{\left(3\right)}\left[i\right] \end{array}$$

where:

(20) $$\begin{array}{ccc} {\mathrm{LSS}}_{l}^{\left(1\right)}\left[i\right] & = & \sum_{n=1}^{N_{l}}\langle z_{l,n,i}\rangle \\ {\mathrm{LSS}}_{l}^{\left(2\right)}\left[i\right] & = & \sum_{n=1}^{N_{l}}\langle z_{l,n,i}\rangle \cdot y_{l,n}\\ {\mathrm{LSS}}_{l}^{\left(3\right)}\left[i\right] & = & \sum_{n=1}^{N_{l}}\langle z_{l,n,i}\rangle \cdot y_{l,n}\cdot y_{l,n}^{T} \end{array}$$

${\mathrm{LSS}}_{l}={\left\{{\mathrm{LSS}}_{l}^{\left(1\right)}\left[i\right],\;{\mathrm{LSS}}_{l}^{\left(2\right)}\left[i\right],\;{\mathrm{LSS}}_{l}^{\left(3\right)}\left[i\right]\right\}}_{i=1,...,I}$ are local sufficient statistics (LSS) of node *l*. Therefore, CSS in node *m* is a linear combination of the LSS of nodes in $R_{m}$. If node *l* has a large number of observations, the accuracy of calculated ${\mathrm{LSS}}_{l}$ should be high and should make an important contribution to the CSS of node *m*. A relatively large $c_{lm}$ in Equation (3) can make this contribution prominent, obtaining accurate estimation of **Θ**.

In the following, we summarize the realization process of the D-MFA algorithm.

Step 1 (Initialization): This initializes the parameters ${\left\{\pi_{i},\mu_{i},A_{i},D_{i}\right\}}_{i=1,...,I}$. Each node *l* broadcasts the number of its observations $N_{l}$ to its neighbors. When receiving this information, each node calculates the combination coefficient by Equation (3).

Step 2 (Computation): Each node *l* in the sensor network computes $\langle z_{l,n,i}\rangle$, $\Omega_{i}$ and $g_{i}$ by Equations (6), (8) and (9), respectively. Then, it computes three local sufficient statistics ${\mathrm{LSS}}_{l}={\left\{{\mathrm{LSS}}_{l}^{\left(1\right)}\left[i\right],\;{\mathrm{LSS}}_{l}^{\left(2\right)}\left[i\right],\;{\mathrm{LSS}}_{l}^{\left(3\right)}\left[i\right]\right\}}_{i=1,...,I}$ according to its own observations $Y_{l}$, by Equation (20).

Step 3 (Diffusion): Each node *l* in sensor networks diffuses its local sufficient statistics ${\mathrm{LSS}}_{l}$, as shown in Figure 1.

Step 4 (Combination): When node *m* (m=1,...,M) receives the local sufficient statistics from all of its one-hop neighbor nodes *l* ($l\in R_{m}$), it computes the combined sufficient statistics $\{{\mathrm{CSS}}_{m}^{\left(1\right)}\left[i\right]$, ${\mathrm{CSS}}_{m}^{\left(2\right)}\left[i\right]$, ${\mathrm{CSS}}_{m}^{\left(3\right)}{\left[i\right]\}}_{i=1,...,I}$ by Equation (19).

Step 5 (Estimation): Node *m* (m=1,...,M) estimates $\pi_{i}$, $\mu_{i}$, $A_{i}$ and $D_{i}$ according to Equations (11), (12), (16) and (17), respectively. Here, we substitute Equation (19) into Equations (11), (12) and (18), reformulating the estimation step as follows:

$$\begin{array}{ccc} \pi_{i} & = & \frac{{\mathrm{CSS}}_{m}^{\left(1\right)}\left[i\right]}{\sum_{i^{\prime }=1}^{I}{\mathrm{CSS}}_{m}^{\left(1\right)}\left[i^{\prime }\right]}\\ \mu_{i} & = & \frac{{\mathrm{CSS}}_{m}^{\left(2\right)}\left[i\right]}{{\mathrm{CSS}}_{m}^{\left(1\right)}\left[i\right]}\\ A_{i} & = & V_{i}g_{i}{\left(g_{i}^{T}V_{i}g_{i}+\Omega_{i}\right)}^{-1}\\ D_{i} & = & \mathrm{diag}\left\{V_{i}-A_{i}\left(g_{i}^{T}V_{i}g_{i}+\Omega_{i}\right)A_{i}^{T}\right\} \end{array}$$

where:

$$V_{i}=\frac{{\mathrm{CSS}}_{m}^{\left(3\right)}\left[i\right]-2{\mathrm{CSS}}_{m}^{\left(2\right)}\left[i\right]\cdot \mu_{i}+{\mathrm{CSS}}_{m}^{\left(1\right)}\left[i\right]\cdot \mu_{i}\mu_{i}^{T}}{{\mathrm{CSS}}_{m}^{\left(1\right)}\left[i\right]}$$

Step 6 (Termination): Node *m* (m=1,...,M) calculates its current local log-likelihood as:

$$\log p\left(Y_{m}|\Theta^{\mathrm{new}}\right)=\sum_{n=1}^{N_{m}}\log\left(\sum_{i=1}^{I}\pi_{i}N\left(y_{m,n}|\mu_{i},A_{i},D_{i}\right)\right)$$

where superscript “new” denotes the newly estimated parameters at the current iteration. If $\log p\left(Y_{m}|\Theta^{\mathrm{new}}\right)-\log p\left(Y_{m}|\Theta^{\mathrm{old}}\right)<\epsilon$, node *m* enters the terminated state; else, go to Step 2 and start the next iteration. It is noted that the terminated nodes do no computation or communication in the following iterations. If one node cannot receive information from a neighbor node in the next iteration, the node will use the received and saved LSS information from that neighbor node at the last iteration when updating CSS. When there is no message communication or information exchange in the network, implying all nodes reach the terminated state, the algorithm ends.

### 3.3. Distributed Density Estimation Algorithm for the M*t*FA

Compared to the MFA, the main difference of the M*t*FA is that it has an additional degree of freedom parameter $\nu_{i}\;\left(i=1,...,I\right)$. Therefore, the parameter set of the M*t*FA to be estimated is $\Theta ={\left\{\pi_{i},\mu_{i},A_{i},D_{i},\nu_{i}\right\}}_{i=1,...,I}$. Moreover, apart from $Z_{l}$ and $U_{l}$, the latent variable $W_{l}={\left\{w_{l,n,i}\right\}}_{i=1,...,I}^{n=1,...,N_{l}}$ should be introduced, explained in Section 2.2. Similarly, for node *m* in a sensor network, a linear combination of local log-likelihoods associated with nodes in $R_{m}$ is defined as:

(21) $$\begin{array}{c} {ℱ}_{m}\left(\Theta \right)=\sum_{l\in R_{m}}c_{lm}\cdot \log p\left(Y_{l}|\Theta \right)=\sum_{l\in R_{m}}c_{lm}\sum_{n=1}^{N_{l}}\log\left(\sum_{i=1}^{I}\pi_{i}\cdot t\left(y_{l,n}|\mu_{i},A_{i}A_{i}^{T}+D_{i},\nu_{i}\right)\right)\; \end{array}$$

The derivation process of the D-M*t*FA algorithm is similar to that of the D-MFA, except that in Step 2, the posterior distribution of $p(w_{m,n,i}|y_{m,n},z_{m,n,i})$ and $p(u_{m,n}|y_{m,n},z_{m,n,i},w_{m,n,i})$ should be computed, and in Step 5, $\nu_{i}$ needs to be estimated. We put this derivation in the Appendix in detail and directly describe the D-M*t*FA algorithm here.

Step 1 (Initialization): This initializes the values of the parameters ${\left\{\pi_{i},\mu_{i},A_{i},D_{i},\nu_{i}\right\}}_{i=1,...,I}$. Each node *l* broadcasts the number of its observations $N_{l}$ to its neighbors. When receiving this information, each node calculates the combination coefficient by Equation (3).

Step 2 (Computation): Each node *l* in the sensor network computes five local sufficient statistics ${\mathrm{LSS}}_{l}=\{{\mathrm{LSS}}_{l}^{\left(1\right)}\left[i\right],\;{\mathrm{LSS}}_{l}^{\left(2\right)}\left[i\right],\;{\mathrm{LSS}}_{l}^{\left(3\right)}\left[i\right]$, ${\mathrm{LSS}}_{l}^{\left(4\right)}\left[i\right],\;{\mathrm{LSS}}_{l}^{\left(5\right)}{\left[i\right]\}}_{i=1,...,I}$ according to its observations $Y_{l}$, given as:

$$\begin{array}{ccc} {\mathrm{LSS}}_{l}^{\left(1\right)}\left[i\right] & = & \sum_{n=1}^{N_{l}}\langle z_{l,n,i}\rangle \\ {\mathrm{LSS}}_{l}^{\left(2\right)}\left[i\right] & = & \sum_{n=1}^{N_{l}}\langle z_{l,n,i}\rangle \cdot \langle w_{l,n,i}\rangle \\ {\mathrm{LSS}}_{l}^{\left(3\right)}\left[i\right] & = & \sum_{n=1}^{N_{l}}\langle z_{l,n,i}\rangle \cdot \langle w_{l,n,i}\rangle \cdot y_{l,n}\\ {\mathrm{LSS}}_{l}^{\left(4\right)}\left[i\right] & = & \sum_{n=1}^{N_{l}}\langle z_{l,n,i}\rangle \cdot \langle w_{l,n,i}\rangle \cdot y_{l,n}y_{l,n}^{T} \end{array}$$

(22) $$\begin{array}{ccc} {\mathrm{LSS}}_{l}^{\left(5\right)}\left[i\right] & = & \sum_{n=1}^{N_{l}}\langle z_{l,n,i}\rangle \cdot \log\langle w_{l,n,i}\rangle \end{array}$$

The expressions of expectations $\langle z_{l,n,i}\rangle$ and $\langle w_{l,n,i}\rangle$ in Equation (22) are given in the Appendix. Moreover, the intermediate variables $\Omega_{i}$ and $g_{i}$ should also be prepared for simplifying expressions in Step 5, shown in the Appendix.

Step 3 (Diffusion): Each node *l* in the sensor network diffuses its local sufficient statistics ${\mathrm{LSS}}_{m}$, as shown in Figure 1.

Step 4 (Combination): When node *m* (m=1,...,M) receives the local sufficient statistics from all of its one-hop neighbor nodes *l* ($l\in R_{m}$), it calculates the combined sufficient statistics, shown as:

(23) $${\mathrm{CSS}}_{m}^{\left(H\right)}\left[i\right]=\sum_{l\in R_{m}}c_{lm}\cdot {\mathrm{LSS}}_{l}^{\left(H\right)}\left[i\right]\;H=1,2,3,4,5$$

Step 5 (Estimation): Node *m* (m=1,...,M) estimates the parameters of the M*t*FA:

(24) $$\begin{array}{ccc} \pi_{i} & = & \frac{{\mathrm{CSS}}_{m}^{\left(1\right)}\left[i\right]}{\sum_{i^{\prime }=1}^{I}{\mathrm{CSS}}_{m}^{\left(1\right)}\left[i^{\prime }\right]} \end{array}$$

(25) $$\begin{array}{ccc} \mu_{i} & = & \frac{{\mathrm{CSS}}_{m}^{\left(3\right)}\left[i\right]}{{\mathrm{CSS}}_{m}^{\left(2\right)}\left[i\right]} \end{array}$$

(26) $$\begin{array}{ccc} A_{i} & = & V_{i}g_{i}{\left(g_{i}^{T}V_{i}g_{i}+\Omega_{i}\right)}^{-1} \end{array}$$

(27) $$\begin{array}{ccc} D_{i} & = & \mathrm{diag}\left\{V_{i}-A_{i}\left(g_{i}^{T}V_{i}g_{i}+\Omega_{i}\right)A_{i}^{T}\right\} \end{array}$$

where:

(28) $$V_{i}=\frac{{\mathrm{CSS}}_{m}^{\left(4\right)}\left[i\right]-2{\mathrm{CSS}}_{m}^{\left(3\right)}\left[i\right]\cdot \mu_{i}^{T}+{\mathrm{CSS}}_{m}^{\left(2\right)}\left[i\right]\cdot \mu_{i}\mu_{i}^{T}}{{\mathrm{CSS}}_{m}^{\left(1\right)}\left[i\right]}$$

In addition, $\nu_{i}$ is updated by solving the following equation:

(29) $$\begin{array}{c} \log\left(\frac{\nu_{i}}{2}\right)-\psi \left(\frac{\nu_{i}}{2}\right)+1-\frac{{\mathrm{CSS}}_{m}^{\left(5\right)}\left[i\right]-{\mathrm{CSS}}_{m}^{\left(2\right)}\left[i\right]}{{\mathrm{CSS}}_{m}^{\left(1\right)}\left[i\right]}-\log\left(\frac{\nu_{i}^{\mathrm{old}}+p}{2}\right)+\psi \left(\frac{\nu_{i}^{\mathrm{old}}+p}{2}\right)=0 \end{array}$$

where ψ(·) is the digamma function and $\nu_{i}^{\mathrm{old}}$ is the value of $\nu_{i}$ on the last iteration of this algorithm. Equation (29) can be solved by some numerical methods, *i.e.*, the Newton method.

Step 6 (Termination): Node *m* (m=1,...,M) calculates its current local log-likelihood $\log p(Y_{l}|\Theta^{\mathrm{new}})$, expressed in Equation (21). The superscript “new” denotes the newly-estimated parameters at the current iteration. The termination condition of the algorithm is the same as that in the D-MFA algorithm.

## 4. Experimental Results

### 4.1. Synthetic Data

**Figure 2:**
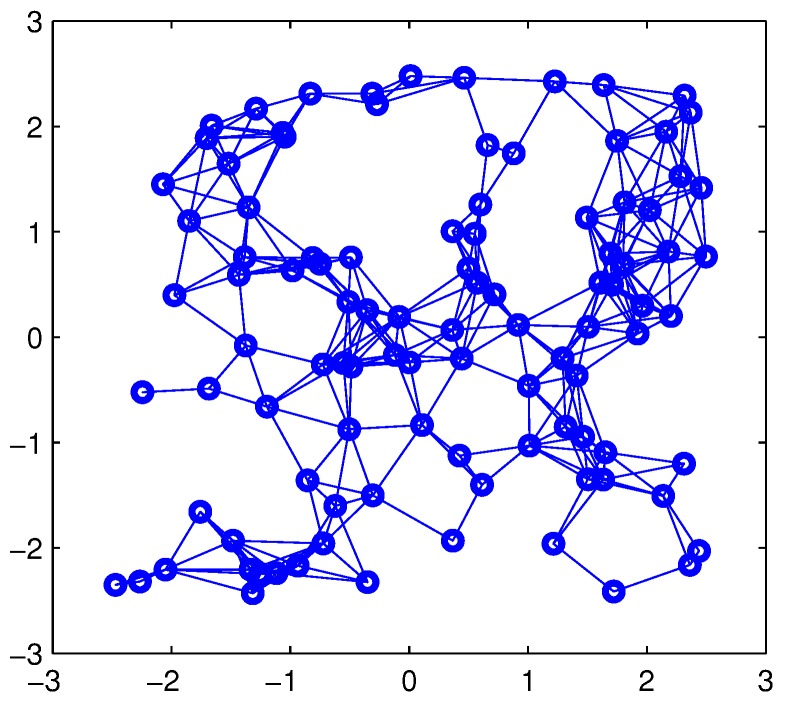
Network connection.

In this subsection, we test the performances of the proposed algorithms on synthetic data. Here, we consider a sensor network composed of 100 nodes to evaluate the estimation performance of the proposed algorithms. Nodes are randomly placed in a square of 5×5. The communication distance is taken as 0.8. In this setting, the connected graph reflecting network topology is shown in Figure 2.

In the first 30 nodes (Node 1–Node 30), each node has 80 observations. In the next 40 nodes (Node 31–Node 70), each node contains 100 observations. In the last 30 nodes (Node 71–Node 100), each node has 120 observations. All of the 10-dimensional observations in the 100 nodes are assumed to be generated from three-component Gaussian mixtures. The parameters are as follows:

$$\begin{array}{c} \left(\pi_{1},\pi_{2},\pi_{3}\right)=\left(0.3,\;0.5,\;0.2\right)\\ \mu_{1}=\left(3\;3\;3\;3\;3\;0\;0\;0\;0\;0\right),\;\mu_{2}=\left(0\;0\;0\;0\;0\;0\;0\;0\;0\;0\right),\\ \mu_{3}=\left(-3\;-3\;-3\;-3\;-3\;\;0\;\;0\;\;0\;\;0\;\;0\right);\\ \Sigma_{1}=\mathrm{diag}(1\;1\;1\;1\;1\;0.1\;0.1\;0.1\;0.1\;0.1),\\ \Sigma_{2}=\Sigma_{1},\;\Sigma_{3}=\Sigma_{1}. \end{array}$$

We adopt several models to represent the distributions of these observations, and the task is to estimate parameters in the models. Here, we compare the performance of four schemes. In the first scheme, the standard EM algorithm for the MFA (S-MFA) is implemented in a centralized unit using all observations from 100 nodes. In the second scheme, the D-MFA algorithm proposed in Section 3.2 performs simultaneously in all nodes. In the third scheme, the EM algorithm for the MFA runs in each node using only local observations of that node. In other words, there is no information exchange among nodes. We abbreviate it as non-cooperation MFA (NC-MFA) for description convenience. In the last scheme, the distributed EM algorithm for the GMM (D-GMM) is implemented. In the D-GMM, the objective function is similar to that of the proposed D-MFA, except that MFA is replaced by GMM. It should be emphasized that the centralized unit is assumed to be always reliable in the S-MFA under ideal conditions. However, this condition is not always fulfilled when the centralized unit fails. Therefore, the S-MFA is seldom adopted in sensor networks. The aim is to test whether the estimation performance of the D-MFA can approach that of the S-MFA.

**Figure 3:**
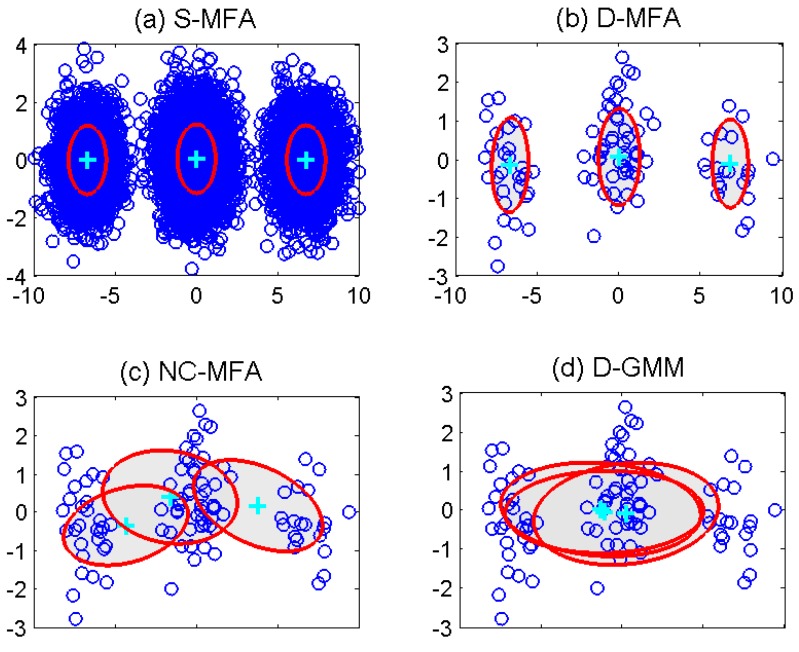
Scatter plot of observations with the estimated parameters at 2D principal subspace using different schemes: (**a**) S-MFA; (**b**) D-MFA; (**c**) NC-MFA; (**d**) D-GMM.

**Figure 4:**
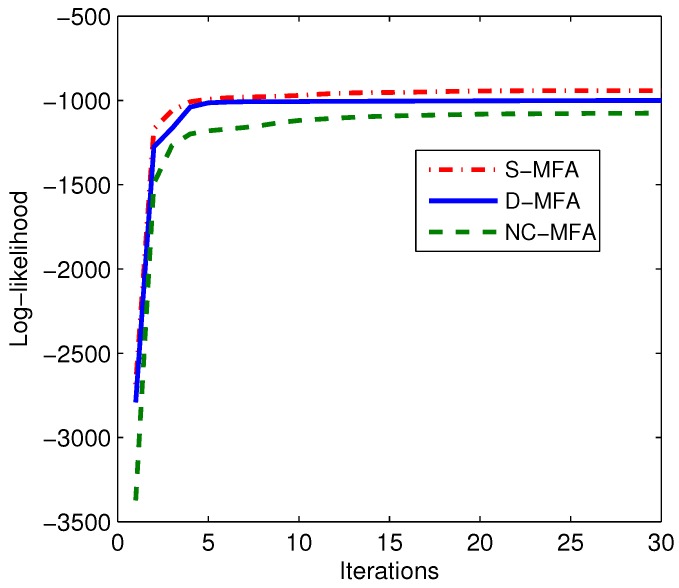
Log-likelihood changes of three MFA schemes during 30 iterations.

In the initialization of these MFA schemes, the dimension of factors are set to five. $\left(\pi_{1}^{0},\pi_{2}^{0},\pi_{3}^{0}\right)=\left(1/3,1/3,1/3\right)$, $\left\{\mu_{1}^{0},\mu_{2}^{0},\mu_{3}^{0}\right\}$ are set as randomly-selected observations in the those nodes. The initial elements in $\left\{D_{1}^{0},D_{2}^{0},D_{3}^{0}\right\}$ and $\left\{A_{1}^{0},A_{2}^{0},A_{3}^{0}\right\}$ are generated by standardized normal distributions. In order to make the estimation results visible, the principal component analysis is performed for observations, obtaining the two largest eigenvalues and the associated eigenvectors. Then, the observations, the estimated means $\mu_{i}\;\left(i=1,2,3\right)$ and the covariances $\Sigma_{i}\;\left(\Sigma_{i}=A_{i}A_{i}^{T}+D_{i}\right)$ after the termination of the algorithms can be projected into 2D principal subspace [34]. Figure 3 illustrates the results of the estimated parameters at the 2D principal subspace in these four schemes. In this figure, the estimated mean $\mu_{i}$ of each component is denoted by “+”, and the estimated covariance $\Sigma_{i}$ is represented by shaded ellipse. Concretely, in Figure 3a, parameters can be correctly estimated by the S-MFA, as the centralized unit can use all of the observations directly. In Figure 3b–d, the results of a randomly-selected node are given. For the NC-MFA, the appropriate parameters are incorrectly estimated, as it can only use its own observations, which also happens in other nodes. For the D-GMM, as it is based on GMM, it cannot describe and process high-dimensional observations well. Finally, in the D-MFA, each node can receive the calculated LSS from nodes in its neighborhood set and combine them for parameter estimation. Compared to GMM, MFA can reflect the properties of these high-dimensional observations more accurately. Therefore, the estimated means and covariances are correct in the D-MFA, as shown in Figure 3b. The other nodes have the same results as this selected node, which are not given here due to space limitation. Moreover, as the same observations and models are used in three MFA schemes, the changes of the average log-likelihood over all nodes in the S-MFA, log-likelihood of the D-MFA and the NC-MFA are shown in different lines in Figure 4. We can see that as the iteration increases, the D-MFA is convergent. Its convergence performance approaches that of the S-MFA.

**Figure 5:**
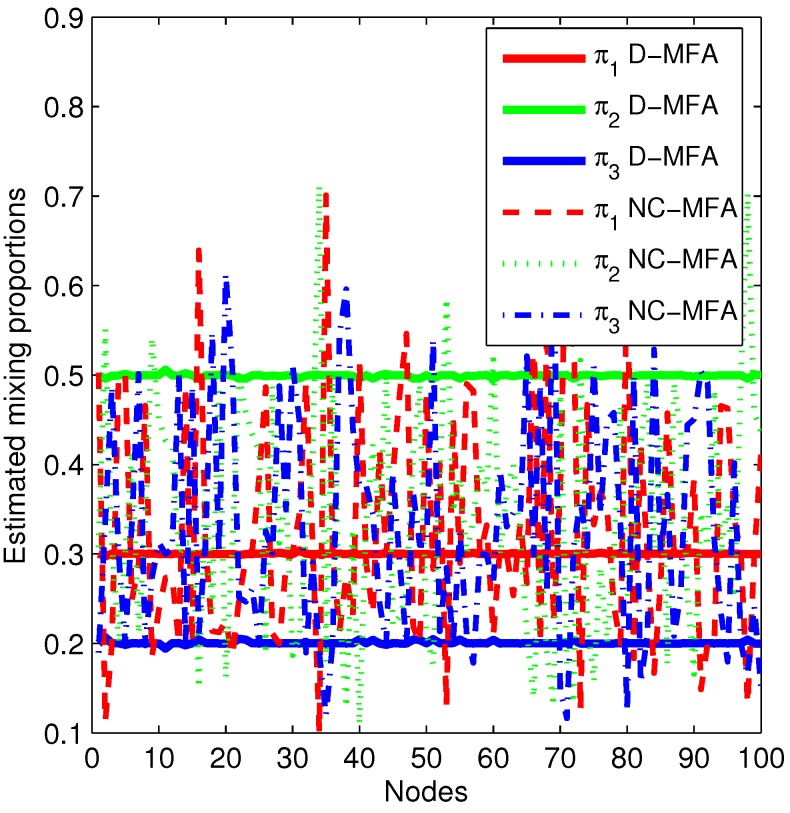
The estimated mixing proportions $\left(\pi_{1},\;\pi_{2},\;\pi_{3}\right)$ in the D-MFA and the NC-MFA at 100 nodes.

**Figure 6:**
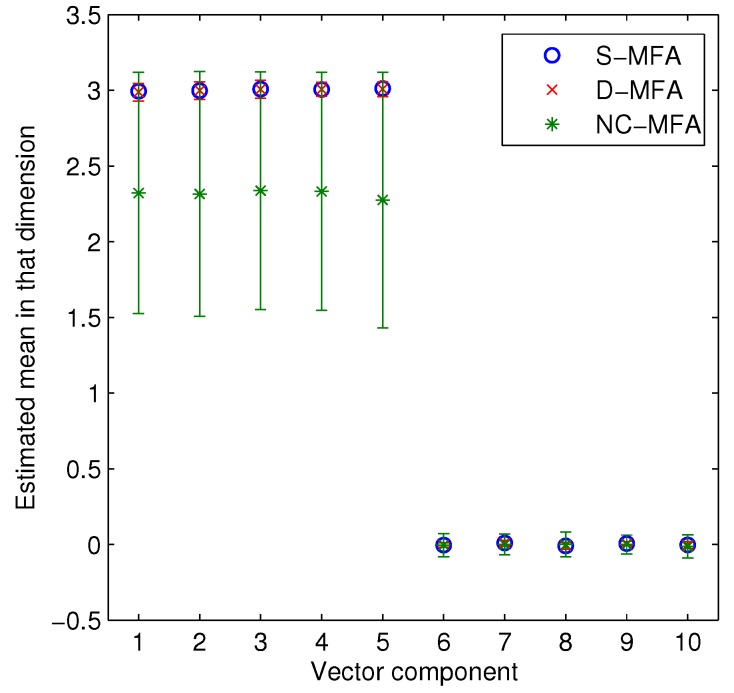
The mean and standard deviation of all of the vector components in estimated $\mu_{1}$ over 100 nodes.

In order to further show the estimation accuracy of the D-MFA at all of the nodes in a sensor network, we select two kinds of parameters $\left(\pi_{1},\pi_{2},\pi_{3}\right)$ and $\mu_{1}$, giving the estimation results of these parameters. In Figure 5, the estimated $\left(\pi_{1},\pi_{2},\pi_{3}\right)$ in the D-MFA and the NC-MFA at all 100 nodes are provided. In the NC-MFA, each node cannot correctly estimate parameters due to limited observations and no information exchange with other nodes, shown by dashed lines. On the contrary, after the D-MFA converges, the estimated values $\left(\pi_{1},\pi_{2},\pi_{3}\right)$ in all 100 nodes approach their true values (0.3,0.5,0.2). In Figure 6, we compare all of the vector components in $\mu_{1}$ estimated by these three MFA schemes. It is noted that for the D-MFA and the NC-MFA, we give the mean and standard deviation of each vector component over 100 nodes to reflect the whole performance of network. We can clearly see that the S-MFA can correctly estimate $\mu_{1}$, as it can use all of the observations. For the D-MFA, the mean of estimated $\mu_{1}$ in each vector component approaches the corresponding true value (3333300000), while the mean of $\mu_{1}$ obtained by the NC-MFA is not consistent with true value. Moreover, the standard deviation of D-MFA is smaller than that of the NC-MFA. Since the other parameters lead to similar results, we omit them here.

**Figure 7:**
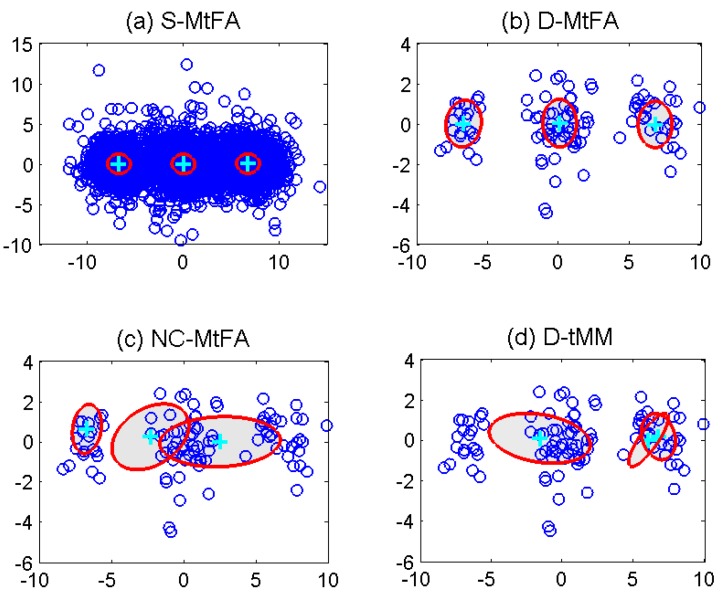
Scatter plot of observations with the estimated parameters at the 2D principal subspace using different schemes: (**a**) S-M*t*FA; (**b**) D-M*t*FA; (**c**) NC-M*t*FA; (**d**) D-*t*MM.

For the D-M*t*FA algorithm, we test its performance and compare it to the S-M*t*FA, the NC-M*t*FA and the D-*t*MM. It is noted that the S-M*t*FA, the NC-M*t*FA and the D-*t*MM can be realized by replacing Gaussian distributions in the S-MFA, the NC-MFA and the D-GMM with Student’s *t*-distributions, respectively. Here, observations are generated by mixtures of Student’s *t*-distributions. The parameters ${\left\{\pi_{i},\mu_{i},\Sigma_{i}\right\}}_{i=1,2,3}$ are unchanged while $\nu_{1}=\nu_{2}=\nu_{3}=5$. In Figure 7, the scatter plot of observations with the estimated parameters at the 2D principal subspace is shown. It is noted that for the D-M*t*FA, the NC-M*t*FA and the D-*t*MM, the results of a randomly-selected node are given. From this figure, we can see several observations located out of ordinary regions, which can be taken as outliers. The S-M*t*FA in the centralized unit, shown in Figure 7a, can grasp the distributions, as it can make use of all observations. On the contrary, the performance of the NC-M*t*FA and the D-*t*MM are bad. The reasons are similar to those of NC-MFA and D-GMM, which have been explained. When implementing the D-M*t*FA, parameters can be accurately estimated, while the property of robustness to outliers is still maintained, as shown in Figure 7b. In summary, the proposed D-MFA and the D-M*t*FA can accurately estimate parameters in a distributed way when each node in sensor network has part of the high-dimensional observations.

### 4.2. Real Data

In several countries, there are monitoring sites located in different regions, whose tasks are to detect nutritional ingredients in the wine samples. These sites form a sensor network, in which each site only communicates with its neighbors and can implement local computations. The wine samples sent to these monitoring sites may belong to different cultivars. Therefore, in each monitoring site, these samples need to be classified, which is good for analyzing in-depth the relationship of nutritional ingredients in the wine and their cultivars. It is certain that the more the references are available for each site, the better the results will be. Therefore, the network and cooperation between sites are required.

In this subsection, we consider the wine cultivar clustering problem as a simulation of the above scenario. The database of this problem is the wine dataset, which is one of the most popular datasets in the UCImachine learning repository [35]. In this wine dataset, 178 samples are collected from a chemical analysis of wines grown in three different cultivars in Italy (the No. 1∼No. 59 samples belong to the first class; the No. 60∼No.130 samples belong to the second class; the No. 131∼No. 178 samples belong to the third class). Each sample has 13 attributes, so the dimension of observations is 13. The sensor network is composed of eight nodes, represented as eight monitoring sites. The average number of nodes in the neighborhood set is two, and the graph is guaranteed to be connected. The average number of samples in each site is 22.

Clustering belongs to the unsupervised learning paradigm in machine learning. When the D-MFA (or the D-M*t*FA) is adopted for clustering, initial values of parameters in the D-MFA are set, which are the same as those in Section 4.1. Then, the corresponding algorithm derived in Section 3.2 (or Section 3.3) is performed. After algorithm converges, an additional computation step based on the estimated parameters **Θ** at node *m* is carried out to obtain $\langle z_{m,n,i}\rangle$ by Equation (6). Finally, the cluster decision for each observation $y_{m,n}$ is:

$${𝒞}_{m,n}={\mathrm{argmax}}_{i=1}^{I}\langle z_{m,n,i}\rangle .\;\;m=1,...M,n=1,...,N_{m}$$

**Figure 8:**
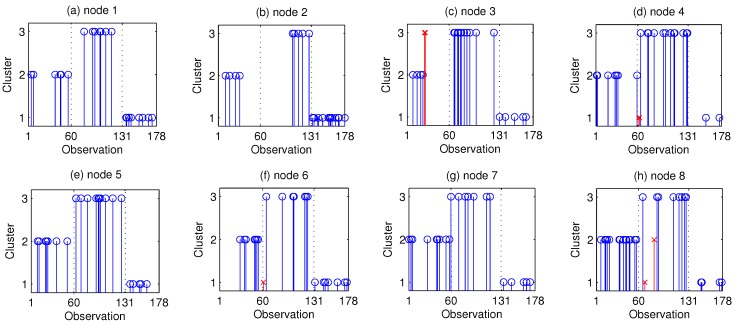
Clustering results of the wine dataset at (**a**–**h**) Node 1∼Node 8.

The clustering results of the D-MFA at Node 1∼Node 8 are shown in Figure 8. In this figure, blue “∘” represents correctly-clustered observations, while red “×” denotes wrongly-clustered ones. From these figures, we can see that the correct ratio in eight nodes are 100%,100%,95.2%,95.5%,100%,95.5%,100%,92.9%. There are five wrongly-clustered observations in all. The correct ratio in the entire network is 97.2%. In order to compare the performance of the D-MFA with that of the S-MFA, we perform the D-MFA and the S-MFA algorithms 20 times. The average correct ratio of these 20 runs by the D-MFA is 96.9%, approaching that by the S-MFA, which is 98.2%. The reason for the small performance gap between the S-MFA and the D-MFA may be that the number of observations for each node is small and the dimension is relatively high. The accuracy of the calculated LSS in Step 2 of the D-MFA are a little worse than those global sufficient statistics obtained by all of the observations in the E-step of the S-MFA. For the NC-MFA, as the number of observations in this example at each node is small, the clustering cannot be implemented. For the D-GMM, as the dimension of observations is high, it also cannot finish the task of this example effectively. Moreover, as there are no outliers in this dataset, the clustering result of the D-M*t*FA is the same as that of the D-MFA, which is not shown here. In summary, we can use the proposed schemes to realize distributed clustering.

## 5. Conclusions

In this paper, we propose a distributed density estimation method base on a mixture of factor analyzers in sensor networks. First, a linear combination of local log-likelihoods associated with nodes in its neighborhood (including itself) is defined as the objective function. In this objective function, the combination coefficients are determined by the number of observations in corresponding nodes. Then, the D-MFA and the D-M*t*FA algorithms are derived. In these algorithms, the combined sufficient statistics of each node are used to estimate parameters, which can be obtained by performing a linear combination of local sufficient statistics from nodes in its neighborhood. Finally, we evaluate the performances of the proposed algorithms and apply them to the tasks of clustering and classification. Experimental results show that they are promising and effective statistical tools for processing the high-dimensional datasets in a distributed way in sensor networks.

In our future work, we will investigate distributed algorithms that can automatically determine the structure of MFA, e.g., the number of components. We also intend to design adaptive strategies to adjust combination coefficients more flexibly. Moreover, the coverage problem [36] in a sensor network is important when implementing distributed algorithms. We will consider this issue in the future.

## Appendix

### A. Derivation of the D-M*t*FA Algorithm

In this Appendix, we give the derivation of the D-M*t*FA after defining the objective function ${ℱ}_{m}\left(\Theta \right)$ in Section 3.3. After introducing three distributions $q(Z_{l})$, $q(W_{l}|Z_{l})$ and $q(U_{l}|W_{l},Z_{l})$, the decomposition shown by Equation (5) holds, where:

$$\begin{array}{c} ℒ\left(q_{l},\Theta \right)=\sum_{Z_{l}}q\left(Z_{l}\right)\int dW_{l}q\left(W_{l}|Z_{l}\right)\int dU_{l}q\left(U_{l}|W_{l},Z_{l}\right)\times \log\left\{\frac{p(Y_{l},U_{l},W_{l},Z_{l}|\Theta )}{q\left(Z_{l}\right)q\left(W_{l}|Z_{l}\right)q\left(U_{l}|W_{l},Z_{l}\right)}\right\} \end{array}$$

$$\begin{array}{ccc} \mathrm{KL}(q_{l}||p_{l}) & = & -\sum_{Z_{l}}q\left(Z_{l}\right)\int dW_{l}q\left(W_{l}|Z_{l}\right)\int dU_{l}q\left(U_{l}|W_{l},Z_{l}\right)\\ & & \times \log\left\{\frac{p\left(Z_{l}|Y_{l},\Theta \right)p\left(W_{l}|Z_{l},Y_{l},\Theta \right)p\left(U_{l}|W_{l},Z_{l},Y_{l},\Theta \right)}{q\left(Z_{l}\right)q\left(W_{l}|Z_{l}\right)q\left(U_{l}|W_{l},Z_{l}\right)}\right\} \end{array}$$

In the first stage, three conditional distributions are needed to compute. Concretely, $p(z_{l,n,i}|y_{l,n})$ at node *l* is:

(A1) $$p\left(z_{l,n,i}|y_{l,n}\right)=\frac{\pi_{i}^{\mathrm{old}}t\left(y_{l,n}|\mu_{i}^{\mathrm{old}},A_{i}^{\mathrm{old}}{\left(A_{i}^{\mathrm{old}}\right)}^{T}+D_{i}^{\mathrm{old}},\nu_{i}^{\mathrm{old}}\right)}{\sum_{i^{\prime }=1}^{I}\pi_{i^{\prime }}^{\mathrm{old}}t\left(y_{l,n}|\mu_{i^{\prime }}^{\mathrm{old}},A_{i^{\prime }}^{\mathrm{old}}{\left(A_{i^{\prime }}^{\mathrm{old}}\right)}^{T}+D_{i^{\prime }}^{\mathrm{old}},\nu_{i^{\prime }}^{\mathrm{old}}\right)}$$

The conditional distribution of $w_{l,n,i}$ given $z_{l,n,i}$ and $y_{l,n}$ is obtained:

(A2) $$p\left(w_{l,n,i}|z_{l,n,i},y_{l,n}\right)=𝒢\left(w_{l,n,i}|\frac{\nu_{i}^{\mathrm{old}}+p}{2},\frac{\nu_{i}^{\mathrm{old}}+{𝒲}_{l,n,i}}{2}\right)$$

where:

$${𝒲}_{l,n,i}={\left(y_{l,n}-\mu_{i}^{\mathrm{old}}\right)}^{T}\left[A_{i}^{\mathrm{old}}{\left(A_{i}^{\mathrm{old}}\right)}^{T}+D_{i}^{\mathrm{old}}\right]\left(y_{l,n}-\mu_{i}^{\mathrm{old}}\right)$$

The expectations $\langle z_{l,n,i}\rangle$ are given by Equation (A1), and $\langle w_{l,n,i}\rangle$ can be obtained from Equation (A2), which is:

$$\langle w_{l,n,i}\rangle =\frac{\nu_{i}^{\mathrm{old}}+p}{\nu_{i}^{\mathrm{old}}+{𝒲}_{l,n,i}}$$

The conditional distribution of $u_{l,n}$ given $w_{l,n,i}$, $z_{l,n,i}$ and $y_{l,n}$ can be computed:

$$p\left(u_{l,n}|w_{l,n,i},z_{l,n,i},y_{l,n}\right)=N\left(u_{l,n}|{\bar{u}}_{l,n,i},\Omega_{i}/\langle w_{l,n,i}\rangle \right)$$

The mean ${\bar{u}}_{l,n,i}$ and $\Omega_{i}$ are:

$$\begin{array}{ccc} {\bar{u}}_{l,n,i} & = & g_{i}^{T}\left(y_{l,n}-\mu_{i}^{\mathrm{old}}\right)\\ \Omega_{i} & = & I_{q}-g_{i}^{T}A_{i}^{\mathrm{old}} \end{array}$$

where:

$$g_{i}={\left[A_{i}^{\mathrm{old}}{\left(A_{i}^{\mathrm{old}}\right)}^{T}+D_{i}^{\mathrm{old}}\right]}^{-1}\cdot A_{i}^{\mathrm{old}}$$

is an intermediate variable introduced to simplify expressions in the following stage. Moreover, the expectations $\langle u_{l,n}\rangle$ and $\langle u_{l,n}u_{l,n}^{T}\rangle$ are:

$$\begin{array}{ccc} \langle u_{l,n}\rangle & = & {\bar{u}}_{l,n,i}\;\;\mathrm{and}\\ \langle u_{l,n}u_{l,n}^{T}\rangle & = & \Omega_{i}/\langle w_{l,n,i}\rangle +{\bar{u}}_{l,n,i}{\bar{u}}_{l,n,i}^{T} \end{array}$$

Similar to the D-MFA, when the first stage finishes, the current lower bound is:

$$\begin{array}{ccc} Q_{m}\left(\Theta \right) & = & \sum_{l\in R_{m}}c_{lm}\sum_{n=1}^{N_{l}}\sum_{i=1}^{I}p\left(z_{l,n,i}|y_{l,n}\right)p\left(w_{l,n,i}|y_{l,n},z_{l,n,i}\right)p\left(u_{l,n}|y_{l,n},z_{l,n,i},w_{l,n,i}\right)\\ & & \times \left\{z_{l,n,i}\log\left[\pi_{i}N\left(u_{l,n}|0,I_{q}\right)𝒢\left(w_{l,n,i}|\nu_{i}/2,\nu_{i}/2\right)N\left(y_{l,n}|\mu_{i}+A_{i}u_{l,n},D_{i}\right)\right]\right\} \end{array}$$

Now, at node *m*, parameters can be obtained by taking derivation of $Q_{m}\left(\Theta \right)$ with respect to **Θ**. First, we obtain:

(A3) $$\pi_{i}=\frac{\sum_{l\in R_{m}}c_{lm}\sum_{n=1}^{N_{l}}\langle z_{l,n,i}\rangle }{\sum_{i^{\prime }=1}^{I}\sum_{l\in R_{m}}c_{lm}\sum_{n=1}^{N_{l}}\langle z_{l,n,i^{\prime }}\rangle }$$

and:

(A4) $$\mu_{i}=\frac{\sum_{l\in R_{m}}c_{lm}\sum_{n=1}^{N_{l}}\langle z_{l,n,i}\rangle \cdot \langle w_{l,n,i}\rangle y_{l,n}}{\sum_{l\in R_{m}}c_{lm}\sum_{n=1}^{N_{l}}\langle z_{l,n,i}\rangle \cdot \langle w_{l,n,i}\rangle }$$

respectively. By substituting Equations (22) and (23) into Equations (A3) and (A4), we can obtain Equations (24) and (25).

Subsequently, by respectively performing derivation of $Q_{m}\left(\Theta \right)$ with respect to $A_{i}$ and $D_{i}$, we have:

(A5) $$A_{i}=\frac{\sum_{l\in R_{m}}c_{lm}\sum_{n=1}^{N_{l}}\langle z_{l,n,i}\rangle \cdot \langle w_{l,n,i}\rangle \left(y_{l,n}-\mu_{i}\right){\langle u_{l,n}\rangle }^{T}}{\sum_{l\in R_{m}}c_{lm}\sum_{n=1}^{N_{l}}\langle z_{l,n,i}\rangle \cdot \langle w_{l,n,i}\rangle \cdot \langle u_{l,n}u_{l,n}^{T}\rangle }$$

and:

(A6) $$\begin{array}{c} D_{i}=\mathrm{diag}\left\{\frac{\sum_{l\in R_{m}}c_{lm}\sum_{n=1}^{N_{l}}\langle z_{l,n,i}\rangle \langle w_{l,n,i}\rangle \left[-A_{i}\langle u_{l,n}u_{l,n}^{T}\rangle A_{i}^{T}\left(y_{l,n}-\mu_{i}\right){\left(y_{l,n}-\mu_{i}\right)}^{T}\right]}{\sum_{l\in R_{m}}c_{lm}\sum_{n=1}^{N_{l}}\langle z_{l,n,i}\rangle }\right\} \end{array}$$

By substituting $\langle z_{l,n,i}\rangle$, $\langle w_{l,n,i}\rangle$, $\langle u_{l,n}\rangle$, $\langle u_{l,n}u_{l,n}^{T}\rangle$ into Equations (A5) and (A6) and by considering the simplified expressions in Equations (22), (23) and (28), we can obtain Equations (26) and (27).

Finally, by performing derivation of $Q_{m}\left(\Theta \right)$ with respect to $\nu_{i}$, the update equation for $\nu_{i}$ is obtained, shown by Equation (29).
